# Sheep Bone Ultrastructure Analyses Reveal Differences in Bone Maturation around Mg-Based and Ti Implants

**DOI:** 10.3390/jfb15070192

**Published:** 2024-07-12

**Authors:** Kamila Iskhakova, D. C. Florian Wieland, Romy Marek, Uwe Y. Schwarze, Anton Davydok, Hanna Cwieka, Tamadur AlBaraghtheh, Jan Reimers, Birte Hindenlang, Sandra Sefa, André Lopes Marinho, Regine Willumeit-Römer, Berit Zeller-Plumhoff

**Affiliations:** 1 Institute of Metallic Biomaterials, Helmholtz-Zentrum Hereon, Max-Planck-Straße 1, 21502 Geesthach, Germany; hanna.cwieka@hereon.de (H.C.); tamadur.albaraghtheh@hereon.de (T.A.); jan.reimers@hereon.de (J.R.); birte.hindenlang@hereon.de (B.H.); sandra.sefa@hereon.de (S.S.); andre.lopes@hereon.de (A.L.M.); regine.willumeit@hereon.de (R.W.-R.); berit.zeller-plumhoff@hereon.de (B.Z.-P.); 2 Department of Orthopaedics and Traumatology, Medical University of Graz, Auenbruggerplatz 5, 8036 Graz, Austria; romy.marek@fhnw.ch (R.M.); uwe.schwarze@medunigraz.at (U.Y.S.); 3 Department of Dental Medicine and Oral Health, Medical University of Graz, Billrothgasse 4, 8010 Graz, Austria; 4 Institute of Materials Physics, Helmholtz-Zentrum Hereon, Max-Planck-Straße 1, 21502 Geesthacht, Germany; anton.davydok@hereon.de

**Keywords:** biodegradable magnesium implants, bone ultrastructure, bone–implant interface

## Abstract

Magnesium alloys are some of the most convenient biodegradable materials for bone fracture treatment due to their tailorable degradation rate, biocompatibility, and mechanical properties resembling those of bone. Despite the fact that magnesium-based implants and ZX00 (Mg-0.45Zn-0.45Ca in wt.%), in particular, have been shown to have suitable degradation rates and good osseointegration, knowledge gaps remain in our understanding of the impact of their degradation properties on the bone’s ultrastructure. Bone is a hierarchically structured material, where not only the microstructure but also the ultrastructure are important as properties like the local mechanical response are determined by it. This study presents the first comparative analysis of bone ultrastructure parameters with high spatial resolution around ZX00 and Ti implants after 6, 12, and 24 weeks of healing. The mineralization was investigated, revealing a significant decrease in the lattice spacing of the (002) Bragg’s peak closer to the ZX00 implant in comparison to Ti, while no significant difference in the crystallite size was observed. The hydroxyapatite platelet thickness and osteon density demonstrated a decrease closer to the ZX00 implant interface. Correlative indentation and strain maps obtained by scanning X-ray diffraction measurements revealed a higher stiffness and faster mechanical adaptation of the bone surrounding Ti implants as compared to the ZX00 ones. Thus, the results suggest the incorporation of Mg^2+^ ions into the bone ultrastructure, as well as a lower degree of remodeling and stiffness of the bone in the presence of ZX00 implants than Ti.

## 1. Introduction

Selecting a material for bone fracture treatment that is beneficial with respect to mechanical, medical, and financial aspects remains a relevant socioeconomic problem [1,2,3]. The materials most used for that purpose, such as titanium (Ti) or steel, often require surgical removal after the fracture is healed. This secondary surgery is not only costly for the healthcare system, but also results in stress and intervention risks to the patient [4]. Biodegradable bone implants are advantageous as they fully degrade in the body and, therefore, help to avoid this secondary surgery.

Among biodegradable materials, magnesium (Mg) appears to be one of the most convenient for the following reasons. Mg is among the elements found in the human body, and it is known to be involved in cell enzymatic reactions, energy metabolism, and cell proliferation [5,6]. Thus, Mg is naturally present in the human body and generally considered biocompatible. Furthermore, the mechanical properties of Mg are suitable for orthopedic applications [7]. For instance, the density and the elastic modulus of the Mg implant are closer to those of bone in comparison to Ti or stainless steel implants [8]. Moreover, controlled Mg degradation under physiological conditions results in harmless degradation products such as Ca(OH)_2_ and Ca- or Mg-based products containing CO_3_ and PO_4_, which can later be absorbed by the physiological environment or removed from the body through the urinal tract [9,10,11,12].

To effectively use Mg-based materials as implants, it is crucial to optimize their degradation rate and mechanical properties. To decrease the degradation rate of Mg and tailor it to correspond to the healing speed of the target tissue, Mg can be alloyed with other elements. For instance, Mg alloyed with calcium (Ca) and zinc (Zn) demonstrates excellent mechanical properties and a desirable degradation rate [7,13]. In parallel, Mg-Ca-Zn alloys offer high biocompatibility as Ca and Zn naturally appear in the body. Zn is involved in numerous biological processes, including the immunological response and bone tissue growth and development. Ca, in turn, is not only one of the main components of bone, but also promotes bone healing [14,15].

ZX00 is a Mg-0.45Zn-0.45Ca (in wt.%) alloy that has demonstrated desirable slow and homogeneous degradation in vivo at rates from 0.08 mm/year to 0.83 mm/year [4,16,17]. Additionally, ZX00 showed adequate biocompatibility and promoted bone tissue formation in a rabbit model [18], and it can promote osteoblast activity in rats in the first ten days of healing [8]. Holweg et al. studied ZX00 implants in sheep and reported that the degradation is not affected by the fracture healing process, with implants being expected to fully dissolve within two years [4].

Martinez et al. studied the degradation behavior of ZX00 screws in sheep in vivo for six, 12, and 24 weeks of healing [19]. The authors revealed that the bone tissue formed in close contact with the implant interface despite gas bubble formation. Complementing the study by Martinez et al., we present a study of the crystal structure and mechanical properties of bone surrounding ZX00 implants in comparison to Ti implants.

Previous studies have shown that the bone ultrastructure can be affected significantly by Mg-based implants. The bone ultrastructure contains collagen fibers and mineralized hydroxyapatite (HAp) crystals deposited between the collagen fibers. Zeller-Plumhoff et al. have reported that the (310) lattice spacing and crystallite size of bone around Mg-Gd alloys are lower than those of bone around Ti implants, while the HAp platelet thickness and collagen orientation were similar [20]. Grünewald et al. reported a decrease in the HAp crystalline order and the platelet thickness in areas with a high Mg concentration, which is caused by Mg incorporation in the bone ultrastructure in the presence of ZX50 and WZ21 implants [20,21]. A similar observation was made by Liebi et al., who reported a decrease in the platelet thickness and degree of orientation of the HAp crystals with increasing distance from ZX10 implants [22]. Meischel et al. have shown that the micro-hardness of the rat bone temporally decreases around WE21 implants and is restored to the initial values after 18 months with the complete degradation of the implants [23]. Grünewald et al. reported an increase in bone hardness around the ZX50 implant with time, revealed using nanoindentation [20].

In the present study, we investigate the influence of ZX00 bone implants on the ultrastructure of the newly formed bone surrounding the degrading implant using the synchrotron radiation scanning small (SAXS) approach and X-ray diffraction (XRD). With the use of SAXS and XRD, one can assess changes in the bone structure down to the nanoscale, with two techniques independently [24]. The samples investigated are ZX00 and Ti explants of screws implanted in sheep tibia. The explants were harvested after 6, 12, and 24 weeks. The sheep model was selected due to the size of the bones and the weight bearing being similar to humans [25]. With the use of SAXS and XRD, it is possible to investigate the d-spacing (lattice spacing), crystallinity, orientation, and the HAp platelet thickness (T-parameter) of the bone. In the case of substitutions of Ca^2+^ by Mg^2+^ in the HAp crystals, a shift of the lattice constant occurs. The inspection of lattice spacing and crystallinity of the HAp crystals by XRD allows the detection of such lattice constant shifts and, therefore, indicates the presence of substitutions. We investigated these parameters as a function of the distance from the bone/implant interface with 0.03 μm resolution. We further performed correlative SAXS/XRD measurements during nanoindentation to reveal differences in the strain distribution in the bone tissue in the vicinity of the bone-to-implant interface. Additionally, bone maturity and osteon density were studied using histology. This paper presents an investigation of bone tissue nanoadaptation with respect to implant interface proximity and strain distribution, and the influence of the Mg-Ca-Zn implants on the bone tissue ultrastructure and maturation is revealed. The combination of the applied techniques enables a better understanding of bone tissue remodeling in the presence of degradable implants as it links information regarding bone ultrastructure, bone maturity, and mechanical response.

## 2. Materials and Methods

### 2.1. Samples

#### 2.1.1. Material Processing

Raw material production and extrusion of 6 mm rods of the alloy ZX00 was carried out at ETH Zürich (Zürich, Switzerland), as published by Holweg et al. [4]. The ZX00 screws (16 mm × 3.5 mm) were manufactured using polycrystalline diamond tools without lubrication, to avoid potential contamination and corrosion attack. After that, the screws were cleaned in an ultrasonic bath with acetone and then dried in a clean-room atmosphere. In the last step, the screws were sterilized by gamma irradiation at a minimum dose of 25 kGy.

#### 2.1.2. Animal Experiments

The animal trial (permit number: BMBWF-66.010-0107-V-3b-2019) was approved by the Austrian Federal Ministry for Science and Research and followed the guidelines on accommodation and care of animals, formulated by the European Convention for the protection of vertebrate animals used for experimental and other scientific purposes. The experiment was performed according to the 3R principles.

In total, twelve three-month-old female lambs were used for this study. The surgical treatment was performed under general anesthesia and sterile clinical conditions at the Medical University of Graz (Graz, Austria). Each animal received three screws into the diaphysis of each tibia, consisting either of ZX00 or Ti. For this study, only the two proximal diaphyseal screws of the left tibia from each animal were used. Using the same implantation region was important for comparability as the ultrastructure varies across different bone sites. For the implantation of the screws, two incisions measuring approximately 2 cm in length were made at distances of 3–5 cm in the medial diaphyseal area. Bicortical drilling was performed with a 2.7 mm drill bit and a 3.5 mm tapper. The Ti screws (16 mm × 3.5 mm) were implanted monocortically into the proximal part, and the ZX00 screws (16 mm × 3.5 mm) were implanted similarly into the mid area of the diaphysis in a strictly lateral direction. A gap of 2 mm was kept between the screw head and the bone, using a spacer. Then, the three animals were euthanized after 6, 12, and 24 weeks, respectively. The harvested tibiae were dehydrated and embedded in Technovit9100 by LLS Rowiak LaserLabSolutions GmbH (Hanover, Germany). Based on previous studies, we performed a power analysis (power: 0.8, error rate: 0.1) yielding a sample size of the three animals [26]. Thus, three animals per time point were used for SAXS/XRD analysis. From each sample, three distinct regions were evaluated, amounting to a total sample size of 12 per group. The in situ SAXS/XRD indentation experiments were limited to one animal per group and one sample per animal, due to the pilot character of the indentation experiments and time restrictions, respectively. The histology was performed for cross-validation; thus, these were also limited to one sample per group, as similar measurements were published by Martinez et al. [19]. An overview of the sample distribution per technique is shown in Table 1.

### 2.2. Scanning SAXS/XRD

For the scanning SAXS/XRD experiments, we manually cut the explant blocks into halves through the screw in the frontal plane. The 0.2 mm diamond band saw (Exakt Saw 300 CL, Exakt Technologies, Inc., Oklahoma City, OK, USA) was used for this purpose. After that, the samples were laser cut into 15 μm thick sections by LLSRowiak LaserLabSolutions GmbH (Hanover, Germany). The sections were then fixed on Kapton tape.

**Figure 1:**
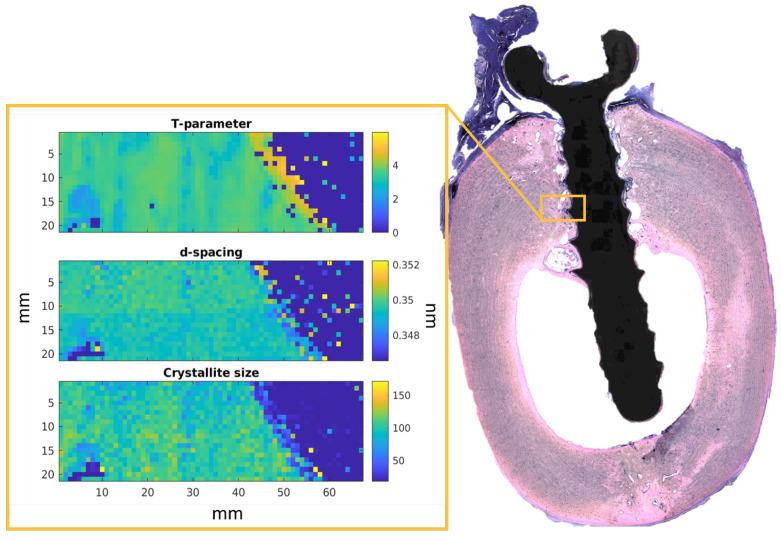
The example of the histological image of the ZX00 sample after 24 weeks of healing. The yellow box represents the scanned region of 0.3 mm by 1 mm^2^. Example maps for one region showing T-parameter, d-spacing, and crystallite size calculated based on (002) Bragg’s reflection distribution are displayed in the yellow frame.

The scanning SAXS/XRD measurements were performed at the P03 microfocus end station at the PETRA III storage ring of the Deutsches Elektronen—Synchrotron (DESY) [27,28]. The energy of the X-ray beam was set at 15 keV with a beam size of 30.6 × 22 μm^2^. To collect the XRD data, the Lambda 9M detector manufactured by X-Spectrum GmbH (Hamburg, Germany) was utilized. For the SAXS measured, a Dectris Pilatus 2M (DECTRIS Ltd., Baden-Daettwil, Switzerland) was placed at 4 m distance from the sample. For the calibration of the XRD data, lanthanum hexaboride (Lab6) was used, while for the SAXS calibration Silver behenate was applied. For each animal, two samples were harvested. Two 1 × 0.3 mm^2^ ROIs with a step size of 27 μm and an acquisition time of 1 s per point were acquired for each sample. An example of the ROI is shown in Figure 1. Complementary data on the T parameter and d-spacing was collected at the I22 beam line at Diamond Light Source (Didcot, UK) [29]. The utilized beam size was 15 μm, and the photon energy was 12.4 keV. The data were collected with 2 s of the exposure time using a Dectris Pilatus 3-2M detector, (DECTRIS Ltd., Baden-Daettwil, Switzerland). The Figure 1 shows a histological image of the explant containing a ZX00 implant 24 weeks after implantation. The ROI is depicted within the orange rectangle. The three maps demonstrate the T-parameter, d-spacing, and crystallite size distribution (green and yellow) in the bone around the implant (blue). In addition to the ROIs, a reference region of the cortical bone further from the implant with the size of 0.3 μm × 0.3 μm was measured. The ROIs were located with the help of an on-axis microscope. In total, four ROIs per sample were measured, resulting in an overall number of 12 regions per material and time point.

### 2.3. Nanoindentation

**Figure 2:**
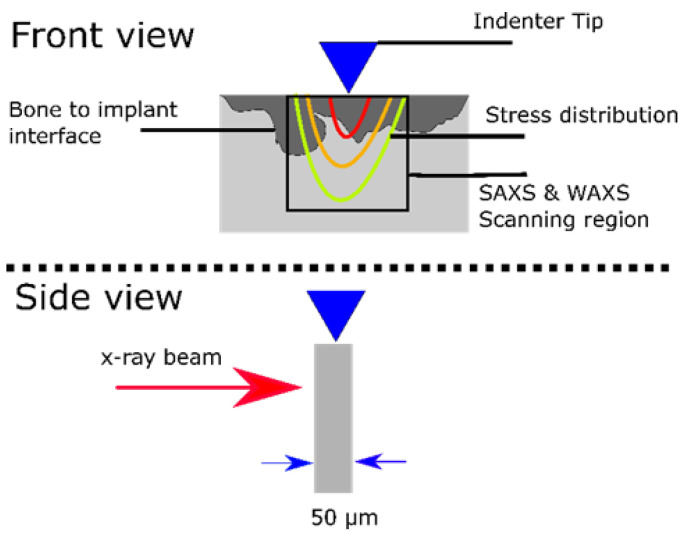
The schematic of the nanoindentation measurement setup for measuring a bone slice (gray) with degradation area (dark gray).

For the in situ nanoindentation study, the embedded bone explants were cut into sections of approx. 100 μm thickness using a Well 3400 diamond wire saw (WELL Diamantdrahtsägen GmbH, Mannheim, Germany) and then fixed onto metal holders. The experiment was performed at the P03 nanofocus end station [28,30] at the PETRA III storage ring of the DESY using their unique indentation setup [31]. The schematic of the measurement is illustrated in Figure 2. A 17 keV photon beam with the size of 1.5 μm × 1.5 μm was used to XRD diffraction rings using a Dectris Eiger 9M (DECTRIS Ltd., Baden-Daettwil, Switzerland). An area of 70 μm × 70 μm under the indenter tip was imaged with an acquisition time of 1 s per scattering image and with a spacing of 2 μm. The region of interest was selected to be at the bone-to-implant interface, while the indenter was pressing the bone at the interface, the force was measured. The scans were obtained after 0 to 2500 mN pressing force with 50–100 mN force steps. Each scan was obtained at a fixed force. One sample per group was assessed.

### 2.4. Data Processing

The PyFAI Python library (ESRF, Grenoble, France) was used for the data reduction and azimuthal averaging processes that are described here. The dead and hot pixels from the SAXS and XRD images were removed by applying a mask. The q-range was calibrated by applying the calibrant data Lab6 obtained during the experiment [32,33]. After that, the processed SAXS /XRD data were azimuthally integrated. The reduced scattering data were analyzed by using a customized processing pipeline in Matlab. The scripts calculate the HAp platelet thickness using the stack-of-cards model [34] by fitting the Kratky plot [35]. For the XRD analysis, the position and full width at half maxima (FWHM) of the HAp diffraction peak (002) were calculated by a Gaussian fit. The (002) Bragg’s peak was selected, as this is a well-isolated peak that is distanced from the Mg or Ti peaks, which can appear on the diffractogram. In order to calculate the d-spacing, Bragg’s law was utilized:

nλ=2dsinθ,

where *n* is the diffraction order, λ is the radiation wavelength, and θ is the glancing angle. To estimate the crystallinity, the Scherrer equation was applied:

$$\tau =\frac{K\lambda }{\beta cos\theta }\;,$$

where *K* is the shape factor close to 1, and β is the fitted Gaussian peak broadening [36]. Then, the d-spacing, T-parameter, and crystallinity were analyzed as a function of the distance from the implant. The qualitative change in the platelet thickness as a function of the distance was determined by fitting a straight line to the T-parameter and calculating the gradient of the fit. The fitting was performed for a distance of up to 0.8 mm from the implant interface. This maximum distance was selected due to data for the ZX00 group after the 12 weeks as this was the maximum distance in that dataset. Crystallite parameters as a function of the distance from the implant are reported in the form of mean and confidence interval.

In order to calculate changes in the strain distribution by indenting the sample, we analyzed the shift of the diffraction peaks as a function of the applied force. We note that we followed a different procedure as described in the literature due to experimental limitations. As samples are exposed to force, the crystals start to deform, and the diffraction ring’s shape becomes an ellipse as the crystal is elongated in one direction and compressed in the perpendicular direction. In the standard approach, the lattice shift of the diffraction peaks, either parallel or perpendicular to the force direction, is monitored as a function of the applied force. However, as bone is not homogeneous even at zero load, a variation in the lattice spacing across the region of interest can be seen. Thus, the exact values from each position have to be extracted and subtracted from consecutive force steps. It cannot always be guaranteed that the registration of the different force steps is perfect. In order to reduce this error, an ellipse was directly fitted to the respective diffraction ring in the reciprocal space. The relative strain was then calculated by

$$\varepsilon_{relative}=a/b$$

with *a* being the semi-minor and *b* semi-major half axis of the ellipse.

This approach has the advantage that the relative change can directly be accessed on the basis of the respective force step in each scan position.

The strain for the ZX00 and Ti samples after six and 24 weeks were compared at a load of 150 μN and 250 μN, respectively. Due to technical issues, the strain in the 12-week samples could not be evaluated with respect to the distance to the indenter tip. The load values were selected as the closest available values for each group. The data were presented in the format of mean values with confidence interval, which was calculated using error propagation.

MATLAB R2021a (The MathWorks, Inc., Natick, MA, USA) computing environment and OriginLab software 2020 (OriginLab Corporation, Northampton, MA, USA) were used for the analysis, statistics, and graphical representation.

### 2.5. Histology

The other halves of the sample blocks were cut and ground into slices of approximately 100 μm thickness, followed by polishing, using the cutting and grinding equipment obtained from EXAKT cutting and grinding equipment (EXAKT Apparatebau, Norderstedt, Germany). These sections were fixed on Methyl methacrylate (MMA) slide samples and afterwards ground, polished, and then stained with Methylene Blue and Basic Fuchsine stainings, following the protocol of Laczo and Levai [37].

Following staining, the samples were scanned with the use of anusing an Olympus BX53 scanning microscope (Olympus, Tokyo, Japan). The qualitative histological analysis was focused on the formation of new bone formation and gas formation. One sample per group was assessed.

Additionally, a quantitative analysis of the bone was performed with the use ofin Adobe Photoshop software 2021 (Adobe Inc., San Jose, CA, USA). We selected two regions of interest (ROIs) per sample were selectedeach. The test area was located in the bone around the implant and the control area in the cortical bone. The ROIs were measured to be around 1.5 mm × 0.5 mm and were segmented into implant, void, osteon, and old bone. Then, the ratio of the osteon area to the rest of the bone area was calculated. One implant per group was analyzed.

## 3. Results

### 3.1. Platelet Thickness (T-Parameter)

**Figure 3:**
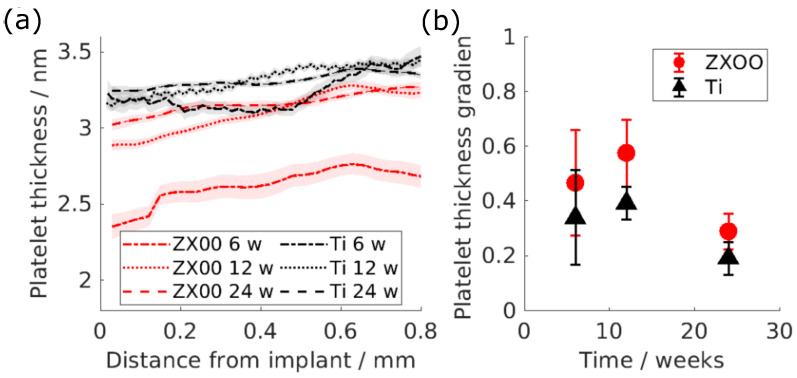
(**a**) Platelet thickness of the HAp as a function of the distance from the implant interface: red is ZX00, black is Ti. The lines represent the mean values, while the shades represent the 95% confidence interval. (**b**) The gradient of the line fitted to the T-parameter as a function of the distance up to 0.8 mm from the implant interface. The error bars indicate the 95% confidence interval of the fit.

Figure 3a demonstrates how the HAp platelet thickness of the bone formed around the ZX00 and Ti implants changes with the distance from the bone-to-implant interface after 6, 12, and 24 weeks of healing. The mean value of the T-parameter was approx. 3 nm. In Figure 3, it can be seen that the platelet thickness of the bone formed around the ZX00 implant tends to decrease in the vicinity of the implant interface, while Ti shows a smaller change.

The slope as a function of the healing time is displayed in Figure 3b for both materials. One can assume that this slope should be close to zero in a homogeneous material and larger than one for material changing from the interface to the bulk. The difference in the gradient between the materials is evident in the graph. The platelet thickness of the HAp around the ZX00 implants after 6, 12, and 24 weeks exhibits a line gradient higher than for Ti, which only shows minor changes with the distance at any given time point.

### 3.2. d-Spacing

**Figure 4:**
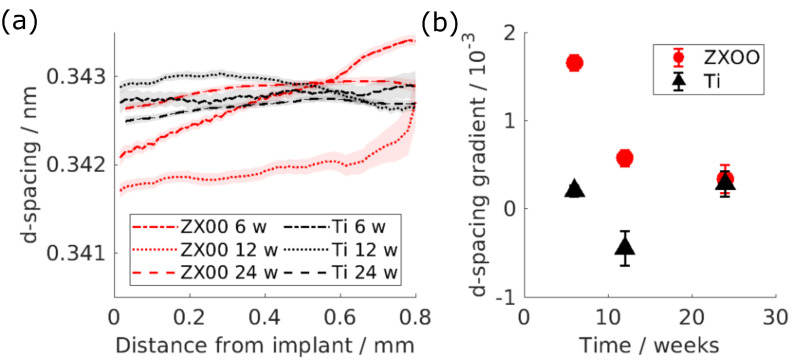
(**a**) Lattice spacing of the HAp as a function of the distance from the implant interface: red is ZX00, black is Ti. The lines represent the mean values, while the shades represent the 95% confidence interval. (**b**) The gradient of the line fitted to the d-spacing as a function of the distance up to 0.8 mm from the implant interface. The error bars indicate the 95% confidence interval of the fit.

Similarly to the T-parameter, the d-spacing was investigated as a function of the distance from the bone-to-implant interface (Figure 4a). Identically to the T-parameter, a prominent change in the d-spacing of the HAp around the ZX00 implants is visible at earlier time points, while it can be regarded as constant or even inverted for Ti samples.

To estimate the degree of that change, the straight line was fitted into the d-spacing at the distance up to 0.8 mm from the implant’s interface and the d-spacing gradient was determined (Figure 4b). The gradient for the HAp d-spacing around the ZX00 implant after 6, 12 and 24 weeks is higher than around the Ti implants.

### 3.3. Crystallite Size

**Figure 5:**
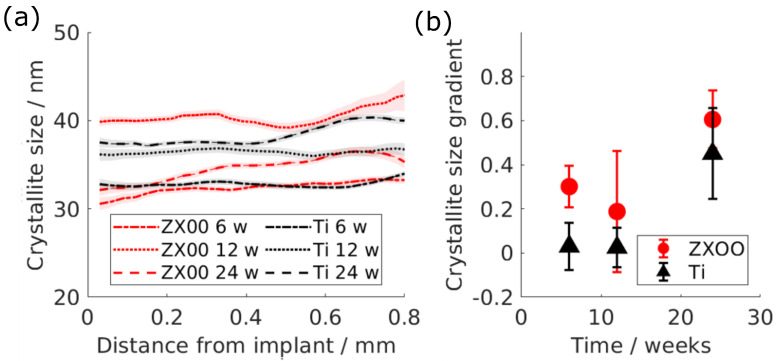
(**a**) Crystallite size of the Bragg’s peak (002) as a function of the distance from the implant interface: red is ZX00; black is Ti. The lines represent the mean values, while the shades represent the 95% confidence interval. (**b**) The change rate of the line fitted to the crystallite size as a function of the distance up to 0.8 mm from the implant interface. The error bars indicate the 95% confidence interval of the fit.

The crystallite size, calculated on the basis of HAp (002) Bragg’s peak as a function of the distance from the implant, is shown in Figure 5a. The crystallite size was measured in an interval of between 30 nm and 40 nm. Contrary to the d-spacing and the T-parameter, the crystallite size does not demonstrate a clear difference between ZX00 and Ti groups.

In order to estimate the change, the gradient of crystallite size as a function of the distance was calculated, which is shown in Figure 5b. The change in the crystallite size does not show a difference between both materials.

### 3.4. Mechanical Testing

**Figure 6:**
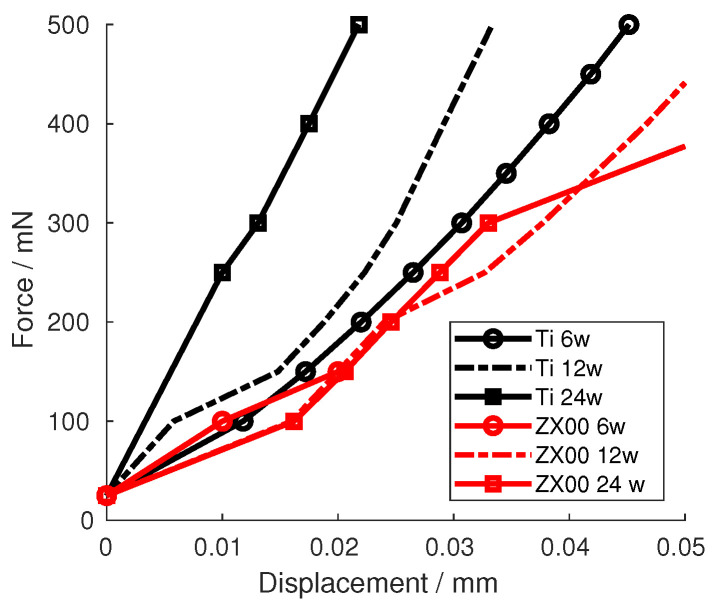
Force–displacement curves of the indenter tip pressing on the Ti and ZX00 interfaces from samples at different healing times.

Figure 6 shows the force–displacement curves of the different Ti and ZX00 samples. The curves show that, after six weeks of healing, the Ti and ZX00 samples exhibit a similar increase in the force after the same displacement. After 12 and 24 weeks of healing, the ZX00 sample shows the same trend as that seen at six weeks. However, Ti shows an increasing steepness in the measured force for the same displacement.

**Figure 7:**
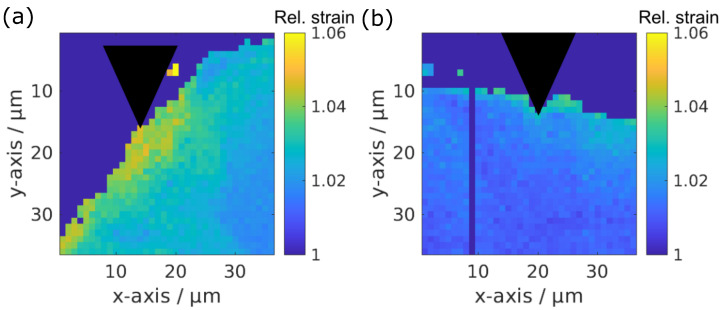
Example maps of the strain distribution in bone around ZX00 (**a**) and Ti (**b**) implants after six weeks of healing. The indenter tip position is indicated by the black triangle.

**Figure 8:**
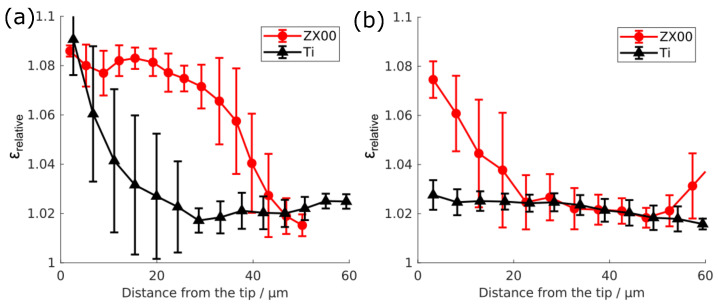
Relative strain $\epsilon_{relative}$ in the bone tissue at the ZX00 or Ti implant interface after six (**a**) or 24 (**b**) weeks after the implantation depending on the distance from the indenter tip at 150 and 200 mN, respectively.

To obtain more detailed insights on the strain distribution on the micrometer scale, a detailed analysis of the strain distribution from the X-ray scattering experiment was performed. Two strain maps are showcased in Figure 7. The change in the strain, depending on the distance from the indenter tip, is depicted in Figure 8 for the bone around the ZX00 and Ti implants after six and 24 weeks of implantation under forces of 150 and 250 mN force, respectively. After six weeks of implantation, the strain is increased for the Ti and ZX00 samples directly at the bone–implant interface. However, Ti shows a strong decay of the strain distribution within a distance of 25 μm. The ZX00 shows a different behavior, exhibiting a high strain up to nearly 40 μm. After 24 weeks of healing, the strain in both samples decreased significantly in comparison to that at six weeks. The Ti sample shows only a slight increase in the vicinity of the interface. ZX00 has a higher strain increase up to 22 μm distance from the tip.

### 3.5. Histology

**Figure 9:**
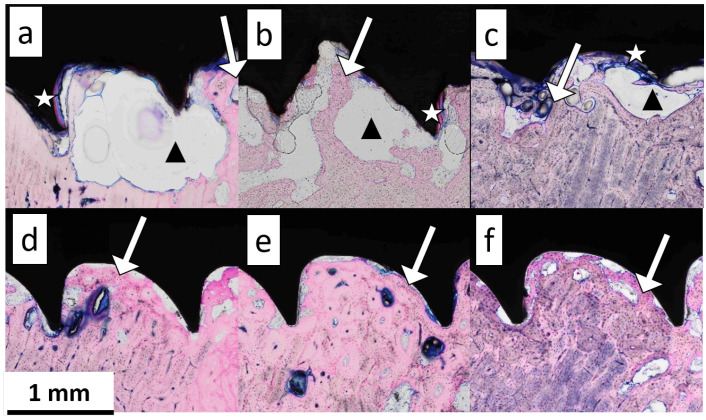
The stained sections of the ZX00 sample after six (**a**), 12 (**b**), and 24 weeks (**c**) and Ti after six (**d**), 12 (**e**), and 24 weeks (**f**). The white stars show the degradation layer, the black triangles show the void area potentially containing gas, and the white arrows point at the newly formed bone tissue.

Figure 9 shows the stained sheep bone slices in the vicinity of the implant with bone tissue stained in pink, soft tissue in blue, and metal implant screws in black. In the regions around the ZX00 screws at 6, 12, and 24 weeks after the implantation (Figure 9a–c), the voids attached to the implants are visible. The voids do not contain any cells. Much smaller voids in comparison to those near ZX00 could be found at the Ti implant surfaces (Figure 9d–f). A large void area, potentially containing gas from the degradation process, was present on the surface of the ZX00 implant after six weeks of healing (Figure 9a, black triangle). After 12 weeks, an increase in the number of void areas distributed on the whole surface was observed (Figure 9b, black triangle). After 24 weeks of healing, a smaller void area was found around the ZX00 screw (Figure 9c, black triangle). The degradation layer forming on the implant interface is visible (white stars) after six weeks of healing. The degradation layer was stained pink due to high Ca and P content, similar to the bone tissue [4]. No fibrosis capsules were observed in any of the samples.

**Figure 10:**
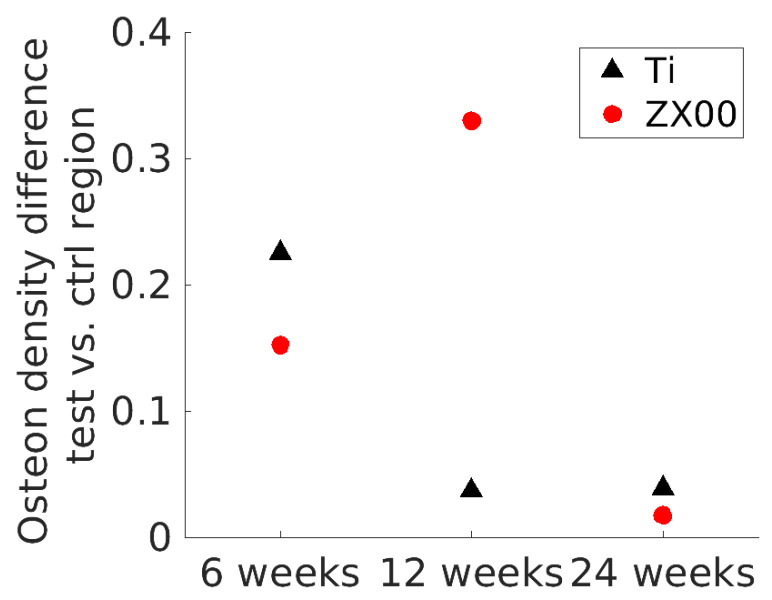
The difference in osteon density of the near-bone ROI and the control region for each group. One sample per material was analyzed.

Newly formed bone tissue is highlighted by the brighter pink staining at the bone-to-implant interface at the bone site in all samples (Figure 9, white arrows). Additionally, the concentration of osteons appears to increase from six to 24 weeks. To derive quantitative data from the histology images, the osteon density around the implant and in the control area were determined. The osteon density around the ZX00 implant was significantly lower than that in the control region at six and 12 weeks of healing, while having a similar value to the control ROI at 24 weeks of healing. For Ti, the dynamics appeared to be different: the osteon density around the Ti implant was significantly lower than that in the control region after six weeks of healing, and did not differ significantly between near-bone ROI and control after 12 and 24 weeks (Figure 10).

## 4. Discussion

The calculated mean platelet thickness of the ovine bone around the ZX00 and Ti implants was in the range of 2.8–3.6 nm. This matches the previously reported values, which range between 2.6 nm and 4 nm, while lower values were reported for the healing bone without the implants [38,39]. The mean values varied between the groups, which is most likely due to differences between individual animals. However, Le Cann et al. showed that platelet thickness increases over time for bone surrounding Ti implants in rabbit tibiae [40]. Thus, the observed differences for ZX00 may be related to ongoing maturation. Le Cann et al. also showed gradients in platelet thickness between peri-implant and cortical bone, similar to the observations in the current study. However, our calculations may be affected by the collagen SAXS signal overlaying the HAp signal, which influenced the Kratky plot and lowered the goodness of the fit to 75%. However, the relative behavior we have observed agrees with the literature. For example, a decrease in platelet thickness closer to the implant was reported previously for WZ21 [23] and ZX10 alloys in rats, which was explained as the bone nanoadaptation to the Mg-based implants. Lower platelet thickness can indicate less mature bone tissue, as the connection between the T-parameter and maturity was observed in other studies [41,42]. By comparison, a study of Mg-xGd alloy implants (x = 5 or 10 wt.%) did not show any changes in platelet thickness; however, it must be noted that much smaller regions of about 100 × 100 μm^2^ were studied [26]. One can also see a slight decrease in the platelet thickness with distance in the Ti group, although it is less prominent. This may be caused by the bone still being immature even around a non-degradable implant after 24 weeks of healing, which was proven by histological staining. A similar effect in rat bone around the Ti implant was reported before [43,44]. It is worth mentioning that the decrease in the T-parameter around the Ti implant was also reported by Bünger et al., and that loading induced by the implant was suggested as a possible reason [45]. Rahmati et al. detected an increase in the platelet thickness in the area around the ZX00 implant in rats up to ten days after implantation, indicating active bone remodeling. Note that the bone tissue response at the earlier stage could differ from the later stage, which was studied in this paper [8].

The computed (002) lattice spacing of 0.3415 nm–0.3435 nm of HAp is similar to the d-spacing reported for the rat bone, which is 0.344 nm [46]. Similar to the T-parameter, the d-spacing of (002) shows a prominent decrease in the area around the ZX00 implant. This result was also seen in the presence of Mg-xGd alloys, where the d-spacing of the (310) HAp plane of the rat bone was lower around the Mg-based implant in comparison to Ti, particularly at early time points [26]. Grünewald et al. reported a decrease in the (002) d-spacing around the ZX50 implant after three months of healing [22]. This effect was accompanied by elevated Mg levels in the bone around the implant. The elevated Mg levels indicate that Mg^2+^ ions are replacing Ca in the HAp crystal structure. The 0.28 lower ionic radius of Mg^2+^ ions in comparison to Ca^2+^ ions leads to a decrease in the lattice spacing around the ZX00 implants [22,47]. In support of this interpretation, Martinez et al. reported traces of Mg in the newly formed bone around ZX00 implants in sheep bone [19].

The crystallite size lies in the range previously reported by other studies, 15–70 nm; the wide range can be explained by the various factors affecting the crystallite size such as sample preparation and age [48,49,50,51]. The crystallite size did not show a significant difference between the groups. It was previously reported that less mature bone tends to have lower crystallinity [52,53], while the lack of difference in crystallite size appears to be in conflict with the observed changes in platelet thickness, it can be explained by different stages in bone maturation and mineralization, while platelet thickness is related to the deposition of not only the mineral hydroxylapatite but also (amorphous) precursors, the crystallite size is related to the growth of the hydroxylapatite phase following the deposition [54,55]. During fracture healing, Dejea et al. have shown that the crystallite size may vary in a nonlinear manner depending on the state of the ongoing remodeling [56]. Thus, the relative similarity in the crystallite size between bone surrounding ZX00 and Ti implants may relate to ongoing mineralization and remodeling. This is in line with our histology results, which indicate that the remodeling process is still taking place around both ZX00 and Ti implants after 24 weeks of healing. It was reported before that elevated levels of magnesium lead to a decrease in the crystallite size of the bone [20,26,57,58]. Mg incorporation around the ZX00 implant, which is evident through the d-spacing and possibly the T-parameter results, might still have an effect on the crystallite size; however, this influence is overshadowed by the effect of bone maturity.

In addition to bone maturity, the histological analysis of the samples might indicate gas formation in the degradation process. The voids were most likely caused by the hydrogen gas formation due to the implant degradation, as the size of the voids around the ZX00 implants was significantly larger in comparison to those around the Ti implants. It was shown that the void area was at the same level at six and 12 weeks; at six weeks, the sample had one large void, and there were several medium-sized voids at 12 weeks. However, as the histological study is two-dimensional, the smaller cavities could still be connected outside the studied slice. After 24 weeks, the void area decreased. This is similar to the results reported before, where the gas formation increased with time, reaching its peak at 12 weeks of healing and then decreasing up to 18 weeks [4,16]. Martinez et al. made the same observation of the gas bubble volumes significantly decreasing between 12 and 24 weeks after implantation of ZX00 implants in sheep [19]. Additionally, Martinez et al. also observed the newly formed bone around ZX00 implants being less mature than that of cortical bone after 24 weeks of healing [19]. Sommer et al. similarly reported the same gas formation volume after six and 12 weeks in the mature rats with a significantly lower gas volume by 24 weeks [17]. On the basis of the volume and surface measurements of the degraded screws after different times of healing presented in the paper [17], it was possible to estimate the degradation rate, which appears to decrease with time. This, in turn, could explain the shrinkage of the gas cavities with time.

The histological analysis showed that the osteon density around the implant at first is significantly lower than compared to the control region. With time, the density around the implant becomes similar to the density at the control region. This happens earlier for Ti (12 weeks of healing) than for ZX00 (24 weeks). The difference might indicate that the bone around Ti matures faster than around ZX00. The lower platelet thickness in the ZX00 group in comparison to the Ti group supports this statement, as it was reported that lower platelet thickness relates to bone immaturity [40]. This may be due to the higher stiffness of Ti implants leading to micro-cracks in the surrounding bone and, therefore, accelerated bone remodeling. A similar observation was reported by Krüger et al., who reported more mature bone around the Ti implant in comparison to Mg-xGd alloys [59]. The correlation between the formed degradation products and bone ultrastructure is not yet thoroughly understood. Some studies such as by Zhang et al. used SEM, EDX, and histology to study Mg-Zn-Mn (Mg—1.0 Zn—0.8 Mn, in wt.%) pins implanted into rat femur to investigate this aspect [60]. They reported that the magnesium phosphate crystalline layer formed on the surface of the Mg-Zn-Mn (Mg—1.0 Zn—0.8 Mn, in wt.%) implant possesses osteoconductive and osteoinductive properties. Kraus et al. employed histology and CT to investigate the osseointegration of ZX50. They suggested that the fast-degrading ZX50 forms an Mg(OH)_2_ layer on the implant surface, which promotes bone formation around it [61]. However, the degradation products were not identified in this study and no comparison with the control group was provided. Contrary to the above-mentioned papers, Chu et al. reported a disruption in bone mineralization around a pure Mg rod in rabbits [62]. With the use of Raman spectroscopy, they detected poorly mineralized HAp as a result of high exposure to Mg. It is reasonable to assume that the bone tissue becomes exposed to increased levels of Mg^2+^ ions during the degradation of the ZX00 implants. This may impact the bone remodeling at the tissue–implant interface and lead to the decreased mineralization or lower maturity level of the bone closer to the ZX00 implant. As the degradation rate slows down with time, so does Mg^2+^ release, shifting the mentioned remodeling and mineralization processes in the bone. This possible interpretation might explain our observations regarding the decrease in the difference in platelet thickness and crystallite size comparing Ti and ZX00, as it is most prominent at the earliest time point, when an increased Mg^2+^ concentration is reasonable. At later stages, the degradation rate and, with it, the Mg^2+^ concentration is lower; thus, the difference in the physiological environment between Ti and Mg which influences bone maturity.

The higher strain increase near ZX00 implants in comparison to Ti implants clearly indicated that the response of the tissue to mechanical stress around the different implants differs, with the bone surrounding ZX00 implants being less stiff. This is corroborated by the force–displacement curves. The nanoindentation analysis of the cortical bone at the bone-to-implant interface demonstrated a lower increase in the strain profile upon deformation at longer healing times as compared to short ones for the Ti implant. Similar observations were made by Bruns et al., who showed a decrease in the strain around Ti implants in rats from four to 12 weeks [63]. They suggested that lower observable strain upon deformation can result from the bone maturing and, therefore, becoming stiffer, which was in agreement with the ultrastructural analysis of the crystallite size [26] and results from histology [59]. This observation can also be made by our analysis, as the force–displacement curves indicate a stiffer matrix around Ti samples after 12 and 24 weeks of healing, and the osteon density presented in this study also hints at a more mature bone at these conditions.

The limitation of the presented study lies in the low amount of samples studied using in situ nanoindentation and histology. However, the presented in situ nanoindentation experiment was carried out as a pilot study, which rationalizes the low sample number. The histological evaluations were performed for the cross-validation and corroboration of the findings regarding the maturity of the bone, obtained from nanoindentation. Importantly, the histological evaluations of the same study type, including the implant material, animal, implantation site, and time points, were published by Martinez et al. [19]. Thus, we believe that meaningful conclusions may be drawn in combination with the existing literature. However, to reach conclusions regarding the absolute mechanical properties, more samples need to be studied in the future to ensure statistical significance and reproducibility. Based on our power analysis, the presented SAXS and XRD studies exhibit a sufficiently large sample size.

## 5. Conclusions

Bone is a hierarchically structured material whose global behavior is also determined by its organization on the ultrastructural level. We have investigated the impact of the magnesium alloy ZX00 on the ultrastructure and mechanical properties.

In summary, this study demonstrates the nanoadaptation in sheep bone caused by biodegradable ZX00 implants and how it depends on the distance from the implant. It was shown that both d-spacing of the HAp (002) plane and the platelet thickness decrease under the influence of ZX00. The former is a result of the Mg^2+^ ions being incorporated into the HAp structure, where they replace Ca^2+^ ions. Crystallinity and platelet thickness, on the other hand, relate to bone remodeling and maturity, which corresponds to larger strains and lower stiffness in the bone surrounding ZX00 implants. This hypothesis is supported by the histology analysis, showing the newly formed bone on the surface of both ZX00 and Ti implants. The lower osteon density in the presence of the ZX00 implants, together with the more prominent decrease in the platelet thickness—closer to the implant surface in comparison to Ti—reveals the slower bone maturation process.

The combination of analysis techniques provides an initial understanding of bone tissue remodeling in the presence of the ZX00 implants, connecting ultrastructure, bone maturity, and mechanical response. These findings enhance the understanding of bone adaptation to implants and can help designing new biodegradable Mg-based implants. To further understand how alterations in the ultrastructure correspond to changes in the mechanical properties of the bone, additional mechanical testing should be conducted in the future.

## Figures and Tables

**Table 1:** The number of scanned regions or ROIs studied per group for each type of characterization technique.

	Methods								
	**SAXS/XRD**			**Nanoindentation**			**Histology**		
time points [weeks]	6	12	24	6	12	24	6	12	24
Ti	12	12	12	1	1	1	1	1	1
ZX00	12	12	12	1	1	1	1	1	1

## Data Availability

The original contributions presented in the study are included in the article, further inquiries can be directed to the corresponding authors.
